# Accurate Cross Sections for Microanalysis

**DOI:** 10.6028/jres.107.041

**Published:** 2002-12-01

**Authors:** Peter Rez

**Affiliations:** Department of Physics and Astronomy and Center for Solid State Science, Arizona State University, Tempe, AZ 85287

**Keywords:** electron beam x-ray microanalysis, electron ionization cross sections, x-ray microanalysis, microanalysis

## Abstract

To calculate the intensity of x-ray emission in electron beam microanalysis requires a knowledge of the energy distribution of the electrons in the solid, the energy variation of the ionization cross section of the relevant subshell, the fraction of ionizations events producing x rays of interest and the absorption coefficient of the x rays on the path to the detector. The theoretical predictions and experimental data available for ionization cross sections are limited mainly to K shells of a few elements. Results of systematic plane wave Born approximation calculations with exchange for K, L, and M shell ionization cross sections over the range of electron energies used in microanalysis are presented. Comparisons are made with experimental measurement for selected K shells and it is shown that the plane wave theory is not appropriate for overvoltages less than 2.5 V.

## 1. Introduction

The intensity of x rays emitted when an electron beam strikes a sample depends on the energy distribution of the electrons in the solid, the energy variation of the ionization cross section of the relevant subshell, the fraction of ionization events that give x rays in the line of interest and the absorption coefficient of the x rays on the path to the detector. This can be summarized as [1]

$$I_{X}=\int_{E_{I}}^{E_{0}}\int I(E,r)\sigma_{X}(E)f_{X}\exp(-\mu_{X}|r_{0}-r|)drdE$$ (1)

where *I*(*E*, ***r***) is the distribution of electrons in the specimen as a function of energy *E* and position ***r***, *σ*_X_(*E*) is the ionization cross section for the relevant subshell, *f*_X_ is the fraction of ionization events producing x rays in the line of interest and *σ*_X_ is the absorption coefficient for the x rays on their path to the detector at position ***r***_0_. The integration is over all electrons that can ionize the subshell of interest, energy *E*_I_, and over the volume of the specimen. For TEM this expression can be considerably simplified as specimens are so thin that energy loss is negligible. (The mean energy loss can actually be measured from the energy loss spectrum which could be recorded at the same time as the x-ray spectrum in suitably equipped microscopes. A typical range of values for the mean energy loss is about 20 eV to 50 eV which is very small compared to the microscope accelerating voltage.) The cross section is then just the ionization cross sections for electrons at the beam energy.

In microanalysis the ionization cross section for production of x rays and the electron energy distribution are often multiplied together to form a new function *Φ*(*ρz*), that is a function of depth in spatially homogeneous specimens [1].

$$I_{X}=\int \phi (r)\exp(-\mu_{X}|r_{0}-r|)dr$$ (2)

$$\phi (r)=\int_{E_{1}}^{E_{0}}I(E,r)\sigma_{X}(E)f_{X}dE$$ (3)

There has been much debate in the literature on the shape of the *Φ*(*ρz*) function [2]. A reliable standardless scheme for determining elemental composition from x-ray intensities requires that all these processes be modeled accurately from the relevant physical theory or values tabulated from experimental measurements.

The energy distribution of electrons in the specimen can be modeled either from numerical solutions of the Boltzmann transport equations [3] or Monte Carlo calculations [4]. Each method has its strengths and weaknesses. The methods that Fathers and Rez [3] used for numerical solution of the Boltzmann transport equation are fast and efficient, but are limited in the number of energy levels that can be used. They can be generalized to layered specimens but are inapplicable for general inhomogeneities. Monte Carlo calculations can be used with arbitrary specimen geometries, but care should be taken that the sampling is done correctly and that simple approximations such as continuous slowing down do not lead to significant error. In other fields such as Medical Physics a combination of small angle transport theory and Monte Carlo calculations is used [5].

The fraction of events giving x rays in the line of interest is a product of the fluorescence yield and the fraction of x rays in a particular line. Reliable calculations for fluorescence yield and x-ray emission rates based on atomic physics are readily available [6,7,8,9]. Absorption coefficients, which could be calculated from first principles expressions for the photoelectric effect, have also been tabulated. Arguably the least known ingredient in the expression for x ray intensity is the electron ionization cross section. Scofield [10] has tabulated K and L shell ionization cross sections for a selection of elements at relativistic energies that are above the energies used in microanalysis. Rez [11] published a number of K shell ionisation cross sections based on hydrogenic wavefunctions, and L and M cross sections based on Hartree-Slater wavefunctions. There have been some calculations for K shells for inert gases and selected transition elements.

In practice microanalysts use cross sections based on the Powell [12] parameterization of the Bethe formula

$$\sigma_{X}({\text{cm}}^{2})=\frac{6.51\times 10^{-14}n_{X}b_{X}}{E_{0}E_{I}}\log_{e}\left(\frac{c_{X}E_{0}}{E_{I}}\right)$$ (4)

where *n_x_* is the number of electrons in the subshell and *b_x_* and *c_x_* are parameters to be determined by fitting to experimental data or other calculation. An obvious shortcoming with this expression is that it fails for electron energies less than *c*_X_*E*_I_ when *c_x_* is greater than 1 (which is usually the case). The same problem arises in the expression for the stopping power and Joy and Luo [13] have proposed a modification for these low energies. My early work [11] was aimed at using first principles ionization calculations to estimate the parameters in the Bethe-Powell expression.

I have now completed systematic calculations of the electron ionization cross sections from all subshells that might be relevant for microanalysis. The plane wave Born approximation with the Ockhur [14] approximation for exchange was used. The lower binding energy limit was determined from the lowest energy x-ray line that could be detected using an ultra thin window or windowless detector, that is about 150 eV. In practice this means L_3_ ionisation cross sections were tabulated from sulfur, and M_5_ cross sections from strontium. The high binding energy limit was set at about 40 kV which meant that K cross sections were calculated up to promethium and all L_3_ and M_5_ cross sections up to uranium were tabulated. The cross sections were calculated for a range of energies from the binding energy or 5 kV, whichever was lower, up to 400 kV. This represents the range of electron energies used in both scanning electron microscopes and transmission electron microscopes. The steps were chosen to correspond to typical step sizes on electron microscopes, though of course intermediate values could be estimated by interpolation.

A selection of the results is presented and they are compared with experimental measurement and other previously reported calculations.

## 2. Theory

The complete theory is given elsewhere [15] so in this paper only the essential points will be summarized. An electron with energy *E* ionizes an inner shell with binding energy *E*_B_, and in the process is scattered by a wave vector ***q*** emerging with energy *E*′ and ejecting an electron of energy *ε* from the atom. Conservation of energy requires that

$$E-E\prime =E_{B}+\varepsilon$$ (5)

Since electrons are indistinguishable there is no way to make a distinction between the scattered electron and the ejected electron so exchange has to be explicitly taken into account. The differential ionization cross section is given by [16]

$$\frac{d^{2}\sigma }{d\Omega d\varepsilon }={\left(\frac{2\gamma }{a_{0}}\right)}^{2}\left[\frac{1}{4}{\left|f+g\right|}^{2}+\frac{3}{4}{\left|f-g\right|}^{2}\right]$$ (6)

where

$$f=\int \int {\Psi^{*}}_{f}(r_{1}){\varphi^{*}}_{f}(r_{2})\frac{1}{\left|r_{1}-r_{2}\right|}\varphi_{i}(r_{2})\Psi_{i}(r_{1})d^{3}r_{1}d^{3}r_{2}$$ (7)

is the “direct” term and

$$g=\int \int {\Psi^{*}}_{f}(r_{2}){\varphi^{*}}_{f}(r_{1})\frac{1}{\left|r_{1}-r_{2}\right|}\varphi_{i}(r_{2})\Psi_{i}(r_{1})d^{3}r_{1}d^{3}r_{2}$$ (8)

is the “exchange” term where the ejected electron appears to have changed places with the scattered electron. The wavefunction *ψ*_i_(***r***_1_) represents the incident electron, *φ*_i_(***r***_2_) the inner shell electron, *ψ_f_* (***r***_1_) the scattered electron and *φ_f_* (***r***_2_) the ejected electron. The constant *a*_0_, the Bohr radius, gives the scale of the interaction and *γ* is the relativistic correction factor. Expanding Eq. (6) gives

$$\frac{d^{2}\sigma }{d\Omega d\varepsilon }={\left(\frac{2\gamma }{a_{0}}\right)}^{2}\left[f^{2}+g^{2}-\frac{1}{2}fg\right]$$ (9)

where the first term in the square brackets is the direct scattering which is much larger than the other two terms.

The incident wave *ψ*_i_(***r***_1_) and the scattered wave *ψ_f_* (***r***_1_) can be represented by plane waves exp(*i****k***_i_***r***) and exp(*i****k***_f_***r***) respectively. The momentum transfer, ***q***, is just the difference between the initial and scattered wavevectors.

$$q=k_{i}-k_{f}$$ (10)

with limits defined by the kinematics of the scattering [17]

$$q_{\min}=\sqrt{k_{i}^{2}+k_{f}^{2}-2k_{i}k_{f}}$$ (11a)

$$q_{\max}=\sqrt{k_{i}^{2}+k_{f}^{2}-2k_{i}k_{f}}$$ (11b)

where *k_f_* the scattered wave vector is

$$k_{f}=\frac{m_{0}c}{\hbar }\sqrt{{\left[\frac{\left(E-E_{B}-\varepsilon \right)}{m_{0}c^{2}}\right]}^{2}+2\left[\frac{\left(E-E_{B}-\varepsilon \right)}{m_{0}c^{2}}\right]}.$$ (11c)

For simplicity just consider the direct term which can be written as

$$\frac{d^{2}\sigma }{d\Omega d\varepsilon }=\frac{4\gamma^{2}}{a_{0}^{2}}\frac{k_{f}}{k_{i}}\frac{{\left|\int \varphi_{f}^{*}(r\prime )\exp(iq\cdot r\prime )\varphi_{i}(r\prime )d^{3}r\prime \right|}^{2}}{q^{4}}$$ (12)

It is more convenient to express the differential cross section in terms of a quantity known as the Generalized Oscillator Strength, *F*(*q*, *ε*) defined by

$$F(q,\varepsilon )=\frac{2m\varepsilon }{\hbar^{2}q^{2}}{\left|\int \varphi_{f}^{*}(r\prime )\exp(iq\cdot r\prime )\varphi_{i}(r\prime )d^{3}r\prime \right|}^{2}$$ (13a)

such that

$$\frac{d^{2}\sigma }{d\Omega d\varepsilon }=\frac{4\gamma^{2}}{a_{0}^{2}}\frac{k_{f}}{k_{i}}\frac{\hbar^{2}}{2m\varepsilon q^{2}}F(q,\varepsilon ).$$ (13b)

To calculate the ionization cross section for the subshell Eq. (13b) has to be integrated over all the allowed wavevectors and over all possible energies of the ejected electron. In our calculations inner shell wave functions were taken from the Dirac-Slater program of Liberman et al. [18], and continuum wavefunctions for the ejected electron were calculated using the self-consistent atomic potential. This potentially laborious computation can be minimized by examining the behavior of the GOS as a function of *q* and *ε*, known as a Bethe surface [17]. In the limit where the ejected electron energy *ε* is about 4 *E*_B_ the ejected electron can be represented by a plane wave with wave vector ***q***. The GOS tends to a Gaussian shape known as the Bethe Ridge with a half width in nm^−1^ approximately given by $35\sqrt{E_{B}}$ where *E*_B_ the binding energy is in Hartrees. The GOS values were tabulated to a momentum transfer of $110\sqrt{E_{B}}$ nm^−1^ over an energy range of 4 *E*_B_. Above this energy the scaled form of the Bethe Ridge was used. It was found that exponential grids in both wave vector and electron energy gave the minimum error in the numerical integration, and 64 points in ***q*** and 64 for *ε* were sufficient to assure numerical convergence to better than 2 %.

An approximate expression for the exchange scattering due to Ochkur [14] also assumes that the ejected electron is a plane wave. The exchange contributions to the scattering can then be formulated in terms of the GOS at the Bethe Ridge, *F*_B_(*q*, *ε*). The GOS in Eq. (13b) is replaced by

$$F\prime =(q,\varepsilon )=F(q,\varepsilon )+\frac{q^{4}}{k_{f}^{4}}F_{B}(q,\varepsilon )-\frac{1}{2}\frac{q^{2}}{k_{f}^{2}}F_{B}(q,\varepsilon ).$$ (14)

This expression only applies when the momentum transfer is very much less than the scattered electron wavevector, which unfortunately does not apply at low energies above threshold. Note also that exchange always reduces the GOS and hence the cross section.

## 3. Results and Discussion

It is well known that the ionization cross section typically rises from threshold to a maximum at about 3 *E*_B_ and then slowly falls off [12,17]. Since this behavior is universal it is often convenient for comparison purposes to plot the cross section against the overvoltage *U*, the ratio of the electron energy to the binding energy.

$$U=\frac{E}{E_{B}}.$$ (15)

**Fig. 1:**
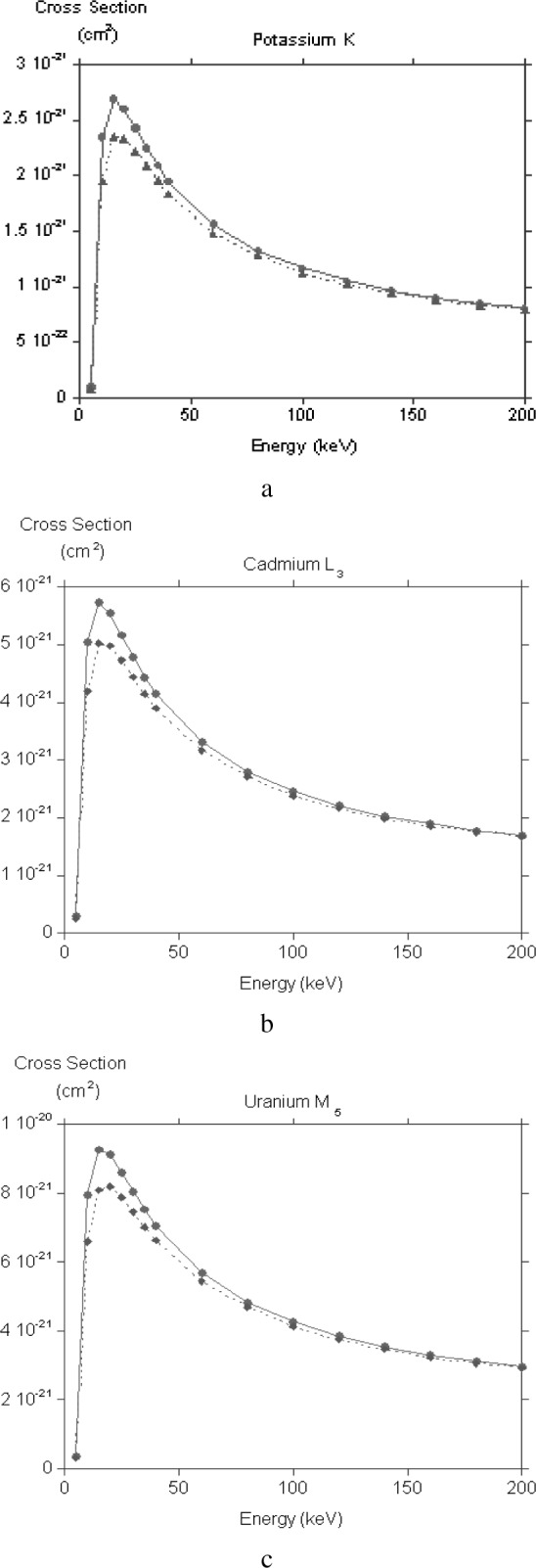
Ionisation cross sections for (a) potassium K, (b) cadmium L_3_ and (c) uranium M_5_. The solid line with filled circles represents the direct contribution, the dashed line with filled diamonds shows the result after taking into account exchange.

In Fig. 1 the cross sections for potassium K, cadmium L_3_ and uranium M_5_ are plotted as a function of electron energy. The dashed curves are the direct contribution only, the solid curves incorporate the effects of exchange with the approximate theory. Exchange not surprisingly has a larger effect near threshold and typically can lower the ionization cross section by 20 % in the region of the peak at about 3 *E*_B_. At higher energies typical of transmission electron microscopes the exchange correction is less important, making a 3 % difference for inner shells with binding energy of about 3.5 kV, and an 8 % difference for inner shells with binding energies of about 11.5 kV. The corresponding figures for a 400 kV accelerating voltage are 1 % and 3 %, respectively.

**Fig. 2:**
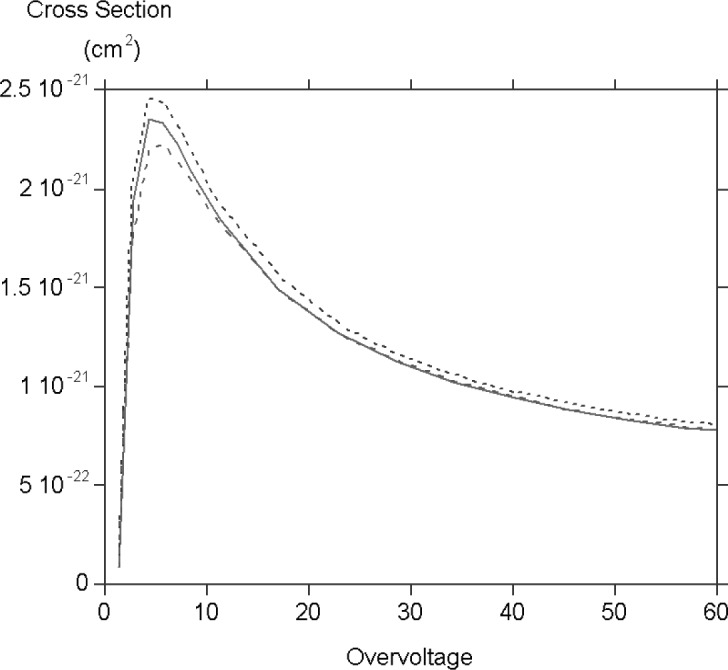
Comparison of ionisation CdL_3_ (short dashed line) and UM_5_ (long dashed line) cross sections scaled to the potassium K (solid line) ionisation cross section. These subshells all have approximately the same binding energy.

Since this is the first time that M cross sections have been explicitly calculated it is interesting to compare them with K and L cross sections for subshells with comparable binding energy. The cross sections for potassium K cadmium L_3_ and uranium M_5_ are all shown as Fig. 2, all scaled to the potassium K cross section. The shapes are very similar though there are some differences in the peak region.

**Fig. 3:**
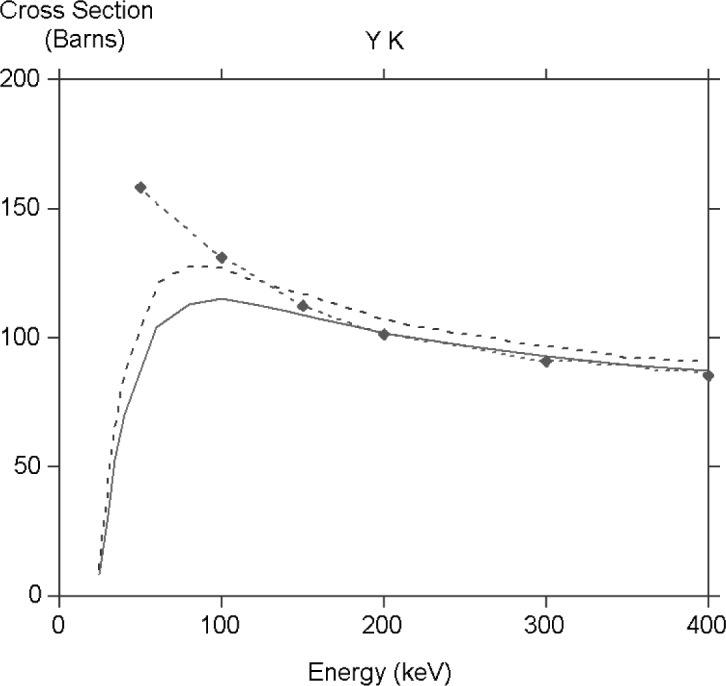
Comparison of the YK calculation with the results of Scofield. The long dashed line is without exchange, the solid line is with exchange, the dashed line with diamonds are Scofield’s results.

There have been very few systematic calculations of cross sections reported in the literature, the most comprehensive being those published by Scofield [10]. He was mainly interested in extreme relativistic energies so there is a very limited overlap with the results presented here. Figure 3 shows a comparison for yttrium K. There is excellent agreement except at the lowest energies where Scofield’s cross section is higher. It is hard to see whether the discrepancy is due to numerical problems as Scofield never shows the interesting region around the peak of the cross section.

**Fig. 4:**
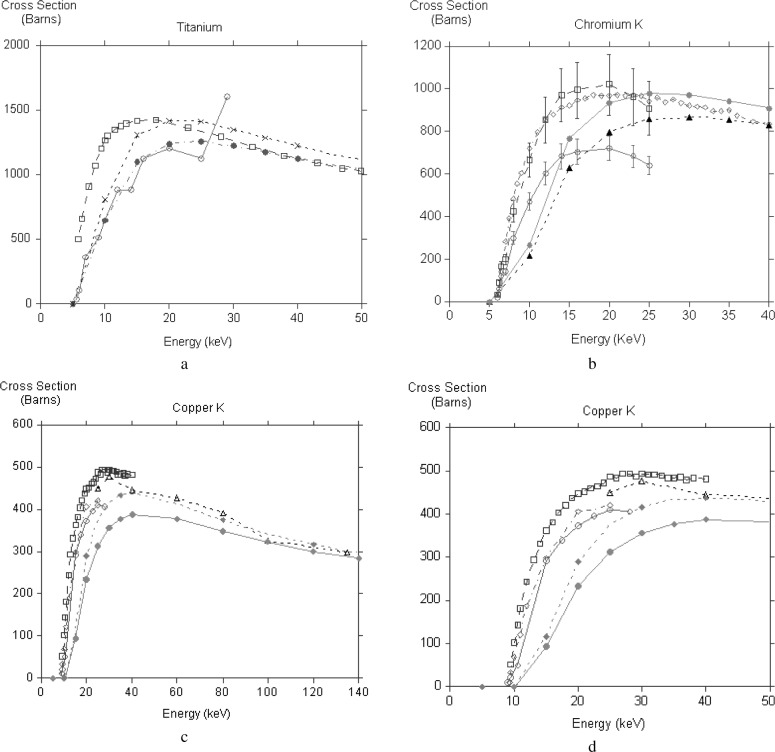
Comparison of experimental results with calculation for Ti, Cr, Cu, and Cu (low energy region).

While comparison with theory is limited to very high energies, the experiments are confined to low energies, very often not even covering the peak of the cross section. Nearly all the results are for K shell ionization of transition element thin films, no doubt because they are easy to prepare. There are additional complications from backscattered electrons generating x rays when the film is lying on a substrate. Luo et al. [19], have used a transport equation treatment to make appropriate corrections to their measurements. The majority of the measurements have been made with dedicated scattering chambers, only the recent work of Llovet et al. [20] made use of a microprobe. In Fig. 4 the present calculations, with and without exchange, are compared to various measurements for TiK, CrK, CuK. There is very little agreement between the experiments for TiK and our calculation lies between the two sets of measurements [21,22]. For CrK Llovet et al. [20] and Luo [19] agree with each other, while He’s results [21] are much lower. The experimental measurements are in broad agreement for copper K, though Llovet et al. [20] is systematically higher than He [21] and Shima [23]. Davis et al. [24] measured cross sections for higher energies and these are in good agreement with our calculations. In the low energy region our calculations for both copper K and chromium K underestimate the cross section in the low energy region and appear to put the peak at too high an energy.

**Fig. 5:**
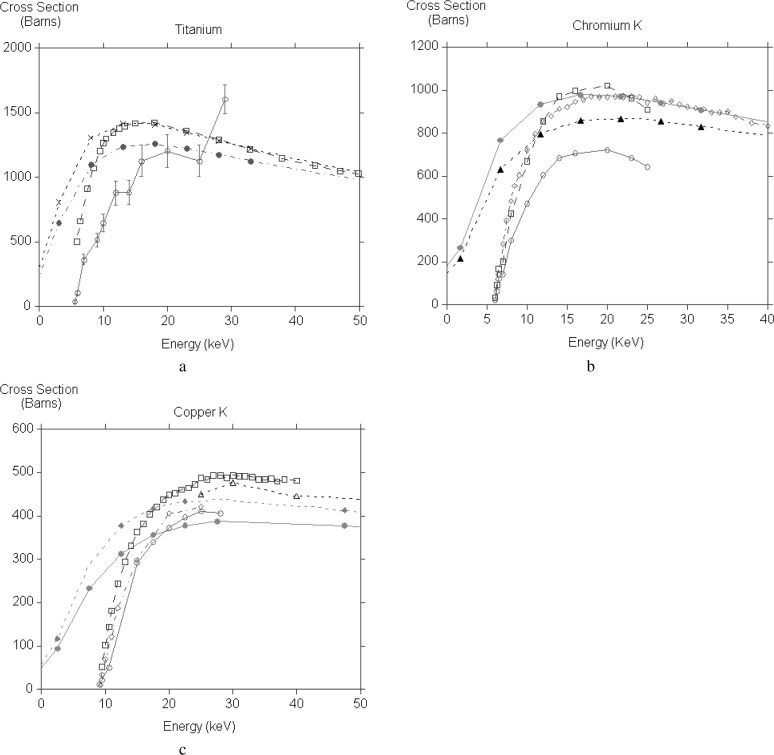
Comparison of experimental results with calculation incorporating acceleration of incident electron for

In his work on the relative cross sections for electron and positron emission Hippler [25] argued that the incident particle was either accelerated or decelerated by the nuclear electrostatic field. He corrected the incident energy by the value of the nuclear electrostatic potential at an average radius for the inner shell wave function. Hippler [25] assumed hydrogenic wavefunctions since he was only interested in K shells. His procedure can be generalized by calculating the average radial value of the wave function explicitly using the wavefunctions calculated by the Dirac-Slater or Hartree-Slater program and then finding the total potential for this point. This potential is then subtracted from the incident energy, which shifts the calculated curves to the left. The comparison with experiment is plotted again as Fig. 5 for the low energy region of tinanium K, chromium K and copper K and although the peak position is now shifted to approximately the correct energy the overall behavior in the low energy region is still incorrect.

The problems arise from an incorrect treatment of the wavefunctions for low energies above threshold. If it is wrong to treat the ejected electron as a plane wave for energies less than 4 *E*_B_, then it is inconsistent to treat the scattered wave as a plane wave at these energies since electrons are indistinguishable. A correct formulation would represent all the electrons as radial solutions of the appropriate energy of the Shrodinger equation with the atomic self-consistent potential. The theory would look very different and the direct them would then be

$$\begin{array}{l} f={\left(4\pi \right)}^{2.5}\left(\frac{a_{0}^{2}}{2}\right)\sum_{\begin{array}{l} l_{4}L_{3}l_{2}k\\ m_{4}m_{3}m_{2}m_{1}q \end{array}}\left(\begin{array}{ccc} l_{4} & k & l_{1}\\ -m_{4} & q & m_{1} \end{array}\right)\left(\begin{array}{ccc} l_{4} & k & l_{1}\\ 0 & 0 & 0 \end{array}\right)\\ \times \left(\begin{array}{ccc} l_{3} & k & l_{2}\\ -m_{3} & q & m_{2} \end{array}\right)\left(\begin{array}{ccc} l_{3} & k & l_{2}\\ 0 & 0 & 0 \end{array}\right)\frac{\sqrt{\left(2l_{4}+1)(2l_{3}+1)(2l_{2}+1)(2l_{1}+1\right)}}{\left(2k+1\right)}\\ \times \left|R_{l_{3}}(\varepsilon ,r_{1})R_{l_{2}}(T-\varepsilon ,r_{2})\frac{2r_{<}^{k}}{r_{>}^{k+1}}j_{l_{2}}(kr_{2})R_{l_{3}}(\varepsilon ,r_{1})dr_{1}dr_{2}\right| \end{array}$$ (16)

where the terms $\left(\begin{array}{ccc} l_{1} & k & l_{2}\\ -m_{1} & q & m_{2} \end{array}\right)$ are Wigner 3*j* symbols and *r*_>_ and *r*_<_ mean the greater or lesser of *r*_1_ and *r*_2_, respectively. The exchange term, *g*, is identical apart from switching *r*_1_ and *r*_2_ in $R_{l_{3}}(\varepsilon ,r_{1})$ and $R_{l_{4}}(T-\varepsilon ,r_{2})$, respectively. This expression is much less amenable to fast computation.

## 4. Conclusions

A selection of results from a comprehensive set of calculations for K, L, and M shell ionization cross sections relevant for microanalysis has been presented. The theory is based on the plane wave Born approximation with the Ochkur [14] expression for exchange. The asymptotic behavior at the Bethe Ridge was used to simplify the calculation and to provide values for the exchange integral.

The cross sections have been tabulated over an energy range from 5 kV to 400 kV appropriate for both scanning and transmission electron microscopes. The cross sections show similar behavior with energy, showing a peak at about 3 *E*_B_. There is good agreement with both Scofield’s [10] calculations and the small number of experimental measurements above this peak. They can therefore be used with confidence in transmission electron microcopy with beam energies of 100 kV and above. The calculations underestimate the value of the cross section in the low energy region and show the position of the peak at too high an energy. This discrepancy is due to the use of a plane wave for the scattered electron. A product of a radial wavefunction and spherical harmonics would be more appropriate for the scattered electron wavefunction and a different theoretical formulation should be used in this region.

## Figures and Tables

**Table:** 

(a)	Ti	long dashed line, open squares solid line, open circles short dashed line, filled circles dash-dotted line, filled circles	Jessenberger and Hink He et al. theory, no exchange theory, with exchange
(b)	Cr	solid line, open circle long dashed line, open square short dashed line, open triangle solid line, filled circle short dashed line, filled triangle	He et al. Luo et al. Llovet et al. theory, no exchange theory, with exchange
(c)	Cu	solid line, open circle long dashed line, open square dash-dotted line, open diamond short dashed line, open triangle short dashed line, filled diamond solid line, filled circle	He et al. Llovet et al. Shima et al. Davis et al. theory, no exchange theory, with exchange
(d)	Cu	low energy region.

**Table:** 

(a)	Ti	long dashed line, open squares solid line, open circles short dashed line, filled circles dash-dotted line, filled circles	Jessenberger and Hink He et al. theory, no exchange theory, with exchange
(b)	Cr	solid line, open circle long dashed line, open square short dashed line, open triangle solid line, filled circle short dashed line, filled triangle	He et al. Luo et al. Llovet et al. theory, no exchange theory, with exchange
(c)	Cu	solid line, open circle long dashed line, open square dash-dotted line, open diamond short dashed line, open triangle short dashed line, filled diamond solid line, filled circle	He et al. Llovet et al. Shima et al. Davis et al. theory, no exchange theory, with exchange
